# Temporary Anchorage Devices and Clear Aligner Therapy: A Systematic Review

**DOI:** 10.7759/cureus.110940

**Published:** 2026-06-16

**Authors:** Mohammed Sawady, Fawaz A Dhayihi, Mohammed S Al Dira, Layla M Arishi, Nawar E Moafa, Ali M Abbas, Ali Y Rajhi, Thekra S Abbas, Rehaf H Madkhali, Ghadi G Jali

**Affiliations:** 1 College of Dentistry, Jazan University, Jazan, SAU

**Keywords:** anchorage control, clear aligners, clear aligner therapy, molar distalization, orthodontic biomechanics, orthodontics, skeletal anchorage, systematic review, temporary anchorage devices, tooth movement

## Abstract

Clear aligner therapy (CAT) has well-documented biomechanical limitations for demanding tooth movements, including molar distalization, posterior intrusion, and significant torque control. Temporary anchorage devices (TADs) provide a compliance-independent source of skeletal anchorage that may help overcome some of these limitations; however, the clinical evidence supporting their combined use with CAT remains incompletely characterized. This systematic review aimed to identify and critically appraise all available clinical evidence on the use of TADs in conjunction with CAT, synthesize findings according to clinical indication, evaluate methodological quality, and identify evidence gaps. Four electronic databases (PubMed, Cochrane Library, Web of Science, and Scopus) were searched from inception to April 2026, with an initial search conducted in May 2025 and an updated search performed in April 2026. Human clinical studies reporting orthodontic outcomes of combined TAD and CAT treatment were eligible for inclusion, while in vitro studies, reviews, and studies involving purely surgical applications of TADs were excluded. Study selection followed the Preferred Reporting Items for Systematic Reviews and Meta-analyses (PRISMA) 2020 guidelines. Methodological quality was assessed using the Joanna Briggs Institute (JBI) Critical Appraisal Checklists for case reports and case series and the Methodological Index for Nonrandomized Studies (MINORS) instrument for comparative and cohort studies. Fourteen studies published between 2009 and 2026, involving 109 patients, met the inclusion criteria, comprising five case reports, three case series, two prospective comparative studies, two retrospective comparative cohort studies, one retrospective observational cohort study, and one retrospective longitudinal study. No randomized controlled trials were identified. Clinical applications included maxillary molar distalization (six studies), mandibular molar distalization (three studies), vertical correction (two studies), transverse scissor-bite correction (one study), impacted canine management (two studies), and complex hybrid or surgery-first approaches (two studies). Across the available comparative studies, TAD anchorage was associated with reduced unwanted side effects, particularly anterior anchorage loss, molar tipping, and vertical adverse effects; however, distalization efficiency remained substantially below planned values, ranging from 32% to 71% in quantitative studies. Case reports and case series provided low-level evidence with substantial inherent bias, while comparative and cohort studies demonstrated moderate methodological limitations. Overall, the combination of TADs and CAT appears technically feasible and demonstrates proof of concept across a range of clinical applications, with the principal benefit being improved control of tooth movement rather than a proportional increase in the magnitude of achieved movement. Nevertheless, the overall certainty of evidence remains low, precluding definitive clinical recommendations. Well-designed randomized controlled trials incorporating standardized three-dimensional outcome measures are needed to establish the effectiveness of this treatment approach.

## Introduction and background

Clear aligner therapy (CAT) has evolved from a treatment option for minor tooth movements into a widely used orthodontic modality capable of managing a broad range of malocclusions [1-3]. Advances in digital treatment planning, thermoplastic materials, and attachment design have expanded its clinical applications, while patient demand for aesthetic, removable appliances has contributed to its growing popularity among both adolescents and adults [2-6].

Despite these advances, CAT remains subject to important biomechanical limitations. Evidence suggests that treatment outcomes often differ from planned tooth movements, particularly for complex movements such as bodily translation, torque control, extrusion, posterior intrusion, and large anteroposterior corrections [1-7]. In contrast, CAT generally demonstrates greater predictability for mild-to-moderate tipping, limited rotations, and arch alignment [4,8]. These limitations are largely attributable to the biomechanical properties of aligners, which primarily generate crown-tipping forces and have limited capacity to deliver the moments required for predictable root movement and three-dimensional control [5-10].

Temporary anchorage devices (TADs), including miniscrews and mini-implants, have become an important source of skeletal anchorage in contemporary orthodontics. By providing anchorage independent of the dentition, TADs facilitate movements such as molar distalization, posterior intrusion, en masse retraction, and impacted tooth traction while minimizing unwanted reciprocal effects [9-14]. The integration of TADs with CAT has therefore emerged as a promising strategy to address some of the biomechanical limitations of aligners and improve control of complex tooth movements.

However, combining TADs with CAT presents unique clinical and technical challenges. Aligner designs must accommodate miniscrews, attachments, and auxiliary force-delivery systems while maintaining fit, comfort, and effectiveness. Furthermore, force application often relies on customized mechanics, including elastics, power chains, cantilever systems, or hybrid approaches that combine removable aligners with fixed auxiliaries [2-7]. As a result, treatment protocols vary considerably across clinicians and clinical indications.

Although the use of TADs with CAT has gained increasing attention, the available evidence remains limited and heterogeneous, consisting largely of case reports, case series, and a small number of comparative studies. Consequently, the effectiveness, predictability, and clinical indications of this combined approach remain incompletely defined.

This systematic review aimed to identify and appraise all available clinical evidence on the use of TADs in combination with CAT, synthesize findings according to clinical indication and tooth movement category, evaluate the methodological quality of the included studies, and identify gaps in the current evidence base and priorities for future research.

## Review

Methodology

Literature Search

This systematic review was conducted in accordance with the Preferred Reporting Items for Systematic Reviews and Meta-Analyses (PRISMA) 2020 guidelines [15]. The review question was structured using the Population, Intervention, Comparison, Outcome (PICO) framework [16]. A comprehensive search of PubMed/MEDLINE, Cochrane Library, Web of Science, and Scopus was performed from database inception to April 2026, with an initial search conducted in May 2025 and an updated search in April 2026. Search terms combined concepts related to CAT and TADs, including miniscrews, mini-implants, and skeletal anchorage. Search strategies were adapted for each database. No restrictions on language, publication date, or study design were applied.

Eligibility Criteria

Studies were eligible if they involved human participants treated with CAT in combination with one or more TADs and reported clinical, cephalometric, or digital model outcomes related to orthodontic tooth movement. All clinical study designs were considered, including case reports, case series, cohort studies, comparative studies, and clinical trials. Studies were excluded if they were in vitro investigations, animal studies, finite element analyses, reviews, editorials, conference abstracts, or studies involving fixed appliances only. Studies in which TADs were used exclusively for surgical stabilization or retention, without an active orthodontic anchorage role during aligner treatment, were also excluded.

Study Selection and Data Extraction

Records were imported into a reference management system, and duplicates were removed. Two reviewers independently screened titles, abstracts, and full-text articles for eligibility. Disagreements were resolved through discussion, with a third reviewer consulted when necessary.

Data extraction was performed independently by two reviewers using a standardized form. Extracted variables included study characteristics, patient demographics, malocclusion type, aligner system, TAD characteristics, treatment mechanics, treatment duration, outcomes assessed, and principal findings. Due to substantial heterogeneity in study design, treatment protocols, outcome measures, and clinical indications, quantitative synthesis was not undertaken. Findings were therefore synthesized narratively and organized according to the primary clinical application or tooth movement category.

Quality Assessment

Methodological quality was assessed independently by two reviewers, with disagreements resolved by consensus or third-reviewer adjudication. Case reports and case series were evaluated using the Joanna Briggs Institute (JBI) Critical Appraisal Checklists [17], while comparative and cohort studies were assessed using the Methodological Index for Nonrandomized Studies (MINORS) instrument [18]. Given the predominance of descriptive studies and heterogeneity of reported outcomes, a Grading of Recommendations Assessment, Development and Evaluation (GRADE) assessment was not performed [19]. Instead, methodological strengths and limitations were summarized narratively. The review was not prospectively registered in an international review registry.

Results

Study Selection

**Figure 1:**
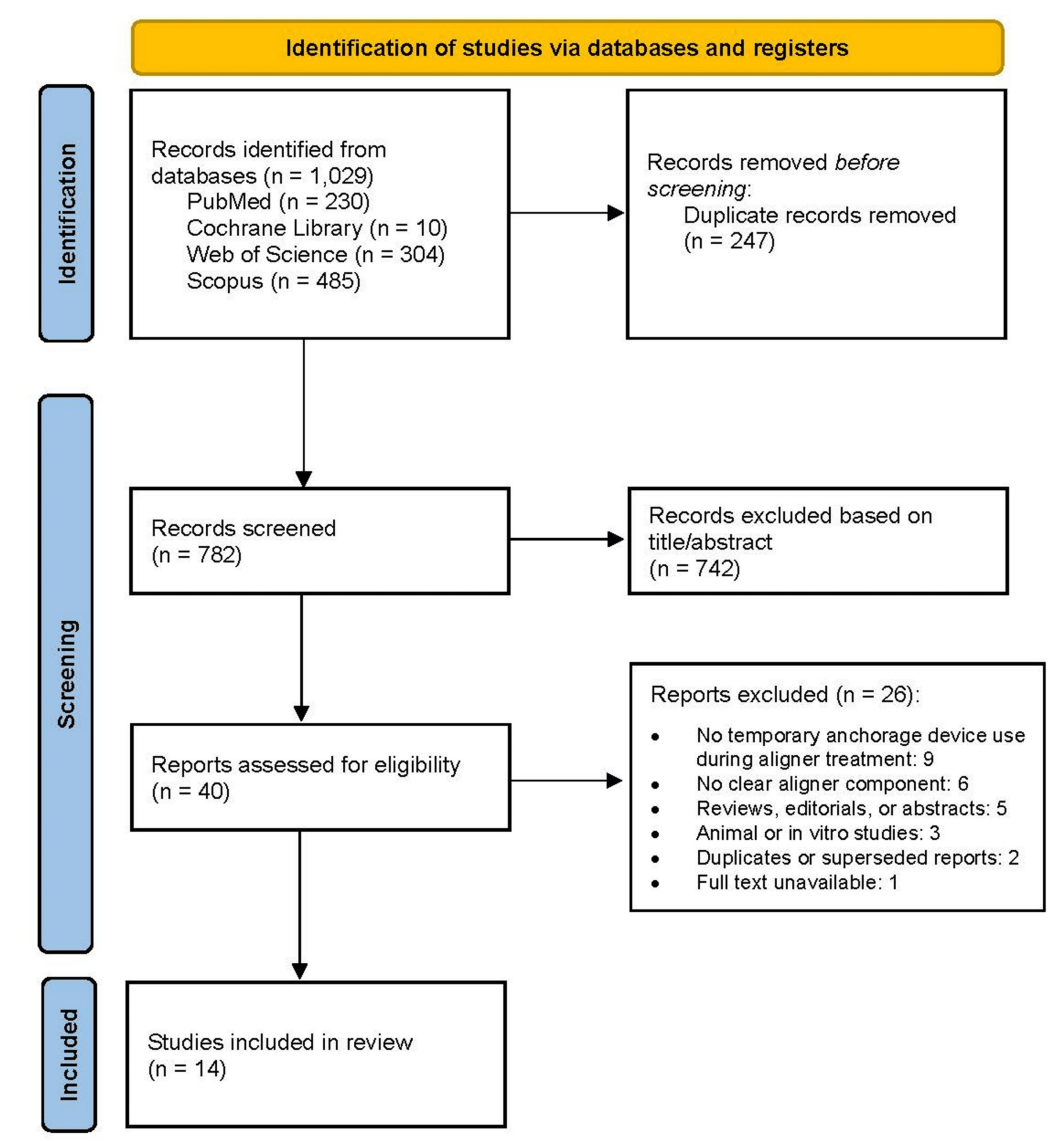
PRISMA flow diagram of study selection process PRISMA: Preferred Reporting Items for Systematic Reviews and Meta-analyses PRISMA flow diagram illustrating the process of study identification, screening, eligibility assessment, and inclusion [15]. Records were identified through database searching and other sources, followed by removal of duplicates. Titles and abstracts were screened, and full-text articles were assessed for eligibility. Studies were excluded at each stage for predefined reasons, resulting in the final number of studies included in the systematic review

The literature search identified 1,029 records, of which 782 remained after duplicate removal. Following title and abstract screening, 40 articles underwent full-text assessment. Twenty-six studies were excluded because they did not involve active TAD use during CAT, lacked a clear aligner component, were nonclinical studies, or had unavailable full texts. One additional study was identified through manual reference screening. Fourteen studies met the eligibility criteria and were included in the review (Figure 1).

Study Characteristics

The 14 included studies comprised five case reports [1,3,5,9,13], three case series [2,4,11], two prospective comparative studies [6,7], two retrospective comparative cohort studies [8,14], one retrospective observational cohort study [10], and one retrospective longitudinal study [12]. No randomized controlled trials were identified (Table 1).

**Table 1:** Characteristics of clinical studies evaluating temporary anchorage devices combined with clear aligner therapy CAT: clear aligner therapy; TADs: temporary anchorage devices; TSADs: temporary skeletal anchorage devices; U: upper; L: lower; U5-U6: upper first and second premolars; L5-L6: lower first and second premolars; U6: upper first molar; U7: upper second molar; L6: lower first molar; L7: lower second molar; IZC: infrazygomatic crest; CBCT: cone-beam computed tomography; FA: fixed appliances; PAR: peer assessment rating index; TMA: titanium-molybdenum alloy; SS: stainless steel; IMPA: incisor mandibular plane angle; SN-OP: sella-nasion to occlusal plane angle; SN-MP: sella-nasion to mandibular plane angle; SNB: sella-nasion-B point angle; SNA: sella-nasion-A point angle; ANB: A point-nasion-B point angle; FMA: Frankfort mandibular plane angle; Wits: Wits appraisal; TMD: temporomandibular disorder; Vivera: Essix-type thermoplastic retainers; CA: clear aligner group; NS: not significant; NOS: not otherwise specified This table summarizes the characteristics of included clinical studies evaluating the use of TADs in combination with CAT. Data include study design, sample size, patient demographics, malocclusion or clinical indication, aligner system, TAD type and location, treatment mechanics, comparators (if applicable), treatment duration, follow-up period, outcomes assessed, and principal conclusions. Additional notes: Treatment protocols and mechanics varied substantially across studies, including staged distalization protocols, elastics (class II, intermaxillary, or intramaxillary), power chains, cantilever mechanics, and hybrid aligner-fixed systems. Due to heterogeneity in study design and outcome reporting, direct quantitative comparisons across studies should be interpreted with caution

Author	Country	Study design	Sample size (TAD-relevant)	Patient age/sex	Malocclusion/clinical indication	Aligner system	TAD/TSAD type	TAD location	Treatment mechanics	Comparator/control	Treatment duration	Follow-up	Main outcomes assessed	Main conclusion
Arveda et al., 2024 [1]	Italy	Case report	1	38-year-old male	Deep bite	F22	Miniscrews	Multiple sites	Hybrid intrusion mechanics	None	9 months	1 month	Overbite reduction	Effective deep bite correction
Capuozzo et al., 2023 [2]	Italy	Case series	2	17–18-year-old females	Impacted maxillary canines	ClinCheck aligners	TADs 8 × 1.5 mm	U5-U6 region	TAD cantilever traction → aligner finishing	None	18-20 months	Retention with Vivera	Canine eruption; periodontal health	Staged TAD traction + aligners achieved successful canine eruption without complications
Choi et al., 2009 [3]	South Korea	Case report	1	16-year-old female	Mild skeletal Class II; bialveolar protrusion; anterior open bite	Duran aligners (0.5-0.75 mm)	Miniscrews 7.0 × 1.8 mm	Buccal alveolar bone U5-U6, L5-L6 bilaterally	Miniscrew-assisted retraction + aligners (6 sets)	None	21 months	16 months retention	Cephalometric changes; anchorage loss; occlusion; stability	Miniscrews + aligners enabled extraction treatment with minimal anchorage loss and profile improvement
Greco and Machoy, 2022 [4]	Italy/Poland	Case series	2	16-year-old male; 43-year-old female	Impacted canines	Aligners	Miniscrews 1.3 × 10 mm	Palatal/buccal	TAD + sectional wires + aligner hybrid approach	None	18 months–2 years	Limited	Occlusion; canine eruption	TAD-assisted aligner systems are effective for impacted canine disimpaction
Iodice et al., 2021 [5]	Italy	Case report	1	21-year-old female	Class II malocclusion; mandibular retrusion; gummy smile	Invisalign	Miniscrews 1.7 × 8 mm	Interradicular maxillary 2.3-2.5	Surgery-first + Invisalign + delayed TAD elastics	None	15 months	End of treatment	Occlusion; skeletal changes; TMD status	Digital surgery-first + aligners + TADs successfully corrected complex class II and gummy smile
Kösen and Aktürk, 2025 [6]	Türkiye	Prospective comparative	22	Young adults	Class II distalization	Invisalign	IZC miniscrews	Infrazygomatic crest	TAD elastics vs Class II elastics	Elastics group	Not specified	Not reported	Ceph; digital models	TADs improved molar distalization and vertical control
Lin et al., 2024 [7]	China	Prospective comparative	17	Adults	Mandibular distalization	Invisalign	Micro-implants	External oblique ridge	TAD elastics vs no TAD	No TAD group	Not reported	Not reported	CBCT distalization	TADs improved mandibular molar distalization efficiency
Liu et al., 2024 [8]	China	Retrospective comparative	30	Adults	Maxillary distalization	Invisalign	Miniscrews	Buccal maxilla	Miniscrew vs Class II elastics	Elastics group	~21-22 months	Not reported	3D movement	Similar efficiency; better anchorage with TADs
Pinho et al., 2024 [9]	Portugal	Case report	1	24-year-old female	Scissor bite	Invisalign	Miniscrews	Palatal/mandibular	Multiscrew + elastics	None	~2.5 years	2 years	PAR index	Stable correction achieved
Pinho et al., 2025 [10]	Portugal	Retrospective cohort	30	Adults	Maxillary distalization	Invisalign	Miniscrews	Multiple sites	Miniscrew-assisted aligners	No miniscrew group	Not reported	Not reported	Model superimposition	Miniscrews improved and achieved distalization
Sabouni et al., 2022 [11]	UAE/India/Egypt	Case series	1	22-year-old female	Open bite	Aligners	Miniscrews	IZC	Posterior intrusion	None	~10 months	Limited	Skeletal changes	TADs enabled mandibular autorotation
Shen et al., 2026 [12]	USA/China	Retrospective longitudinal	21	28.7 ± 4.2 years	Maxillary distalization	Invisalign	IZC TSADs	Infrazygomatic crest	Power chain from premolar to TSAD	No control	~15 months	Not reported	CBCT; accuracy	Limited distalization efficiency (~32-37%) but improved intrusion
Wang et al., 2024 [13]	China	Case report	1	19-year-old male	Class II div 2; ectopic canine	Invisalign	Miniscrew	U5-U6 interradicular	Elastic-assisted distalization + aligners	None	19 months	2 years 3 months	CBCT; occlusion	TAD-assisted aligners improved asymmetric molar distalization
Wang et al., 2024 [14]	China	Retrospective cohort	46	Adults	Class I/II distalization	Clear aligners	Miniscrews (IZC + buccal shelf)	Maxilla/mandible	Miniscrew-assisted distalization vs FA	Fixed appliance group	~33-34 months	Not reported	Ceph; distalization	Similar distalization; better vertical control with aligners

A total of 109 patients were included. The principal clinical applications were maxillary molar distalization (six studies) [6,8,10,12-14], mandibular molar distalization (three studies) [7,10,14], vertical correction (two studies) [1,11], impacted canine management (two studies) [2,4], transverse scissor-bite correction (one study) [9], anterior retraction (one study) [3], and surgery-first or complex hybrid treatment (one study) [5].

Methodological Quality

Overall methodological quality was low to moderate. Case reports and case series provided descriptive clinical data but were limited by the absence of control groups and high susceptibility to selection bias. Comparative and cohort studies demonstrated moderate methodological quality but were limited by small sample sizes, lack of randomization, absence of blinded outcome assessment, and potential confounding. No study was considered to provide high-certainty evidence (Tables 2-4).

**Table 2:** JBI critical appraisal of included case reports studies JBI: Joanna Briggs Institute; RoB: risk of bias This table presents the results of the JBI) Critical Appraisal Checklist for case reports included in the systematic review [17]. Each domain was assessed as “Yes,” “No,” or “Partly” based on reporting completeness and methodological adequacy. Domains include demographic reporting, patient history and timeline, clarity of clinical condition, diagnostic evaluation, intervention description, post-intervention outcomes, reporting of adverse events, and clarity of takeaway lessons. Overall quality and RoB were judged based on a structured qualitative assessment, while the evidence level reflects the inherent limitations of case report designs within evidence hierarchies

Study ID	Demographics	History/timeline	Clinical condition	Diagnostic tests	Intervention described	Post-intervention	Adverse events	Takeaway lessons	Overall quality	Overall RoB	Evidence level
Arveda et al., 2024 [1]	Yes	Yes	Yes	Yes	Yes	Yes	Partly	Yes	Good	Moderate-High	Low
Choi et al., 2009 [3]	Yes	Yes	Yes	Yes	Yes	Yes	Partly	Yes	Good	Moderate-High	Low
Iodice et al., 2021 [5]	Yes	Yes	Yes	Yes	Yes	Yes	Partly	Yes	Good	Moderate-High	Low
Pinho et al., 2024 [9]	Yes	Yes	Yes	Yes	Yes	Yes	Yes/partly	Yes	Good	Moderate-High	Low
Wang et al., 2024 [13]	Yes	Yes	Yes	Yes	Yes	Yes	Partly	Yes	Good	Moderate-High	Low

**Table 3:** JBI critical appraisal of included case series studies CAT: clear aligner therapy; JBI: Joanna Briggs Institute; RoB: risk of bias; TADs: temporary anchorage devices; N/A: not applicable This table presents the results of the JBI Critical Appraisal Checklist for included case series studies within the systematic review evaluating TADs in combination with CAT [17]. Each item of the checklist was assessed and reported as “Yes,” “No,” “Partly,” or “Unclear,” based on the level of methodological reporting and adherence to JBI criteria. The domains assessed include clarity of inclusion criteria, reliability and validity of condition measurement, consecutive and complete inclusion of participants, adequacy of demographic and clinical reporting, completeness of outcome reporting, description of study site, and appropriateness of statistical analysis where applicable. Overall quality, overall risk of bias (RoB), and evidence level were assigned based on methodological rigor and inherent limitations of case series designs. Additional note: Case series inherently provide low-level evidence due to a lack of control groups, limited ability to infer causality, and susceptibility to selection and reporting bias

Study ID	Inclusion criteria	Condition measured reliably	Valid methods	Consecutive inclusion	Complete inclusion	Demographics reported	Clinical info reported	Outcomes reported	Site info	Statistics	Overall quality	Overall RoB	Evidence level
Capuozzo et al., 2023 [2]	Partly	Yes	Yes	Unclear	Unclear	Yes	Yes	Yes	Partly	N/A	Moderate-good	Moderate-high	Low
Greco and Machoy, 2022 [4]	Partly	Yes	Yes	Unclear	Unclear	Yes	Yes	Yes/partly	Partly	N/A	Moderate	Moderate-high	Low
Sabouni et al., 2022 [11]	Partly	Yes	Yes	Unclear	Unclear	Yes	Yes	Yes	Partly	N/A	Moderate	Moderate-high	Low

**Table 4:** Minors assessment of comparative and noncomparative clinical studies MINORS: Methodological Index for Nonrandomized Studies; ROB: risk of bias This table presents the methodological quality assessment of included comparative and noncomparative clinical studies using the MINORS tool [18]. Each item is scored as 0 = not reported, 1 = reported but inadequate, or 2 = reported and adequate. The MINORS tool includes 12 items for comparative studies (maximum score = 24) and 8 items for noncomparative studies (maximum score = 16). Higher total scores indicate better methodological quality. The final columns summarize the total score, maximum possible score, overall interpretation, and overall ROB

Author	Design	1. Aim	2. Consecutive	3. Prospective	4. Endpoints	5. Unbiased	6. Follow-up	7. Attrition	8. Sample size	9. Control	10. Contemporary	11. Baseline equiv.	12. Statistics	Total	Max	Interpretation	Overall ROB
Kösen and Aktürk, 2025 [6]	Prospective comparative	2	1	2	2	1	2	0	2	2	2	1	2	19	24	Moderate-good	Moderate
Lin et al., 2024 [7]	Prospective comparative	2	1	2	2	1	2	1	0	2	2	1	2	18	24	Moderate-good	Moderate
Liu et al., 2024 [8]	Retrospective comparative	2	1	0	2	1	2	1	0	2	2	1	2	16	24	Moderate	Moderate
Pinho et al., 2025 [10]	Retrospective cohort	2	1	0	2	1	2	1	0	1	2	1	2	15	24	Moderate	Moderate
Shen et al., 2026 [12]	Retrospective noncomparative	2	1	0	2	1	2	1	2	-	-	-	-	11	16	Moderate	Moderate
Wang et al., 2024 [14]	Retrospective comparative	2	1	0	2	1	2	1	2	2	2	2	2	19	24	Moderate-good	Moderate

Maxillary Molar Distalization

Maxillary molar distalization was the most frequently investigated indication [6,8,10,12-14]. Across quantitative studies, achieved distalization was consistently lower than planned movement, with reported efficiencies ranging from approximately 32% to 44% [8,10,12].

The available comparative evidence suggests that TAD-supported mechanics improve movement quality rather than substantially increasing distalization magnitude. Studies comparing TAD anchorage with class II elastics reported reduced anterior anchorage loss, less molar tipping, and improved vertical control [6,8]. Similarly, miniscrew-assisted distalization with CAT demonstrated less molar tipping and better vertical control than fixed appliances despite comparable distalization amounts [14]. Additional evidence suggested that infrazygomatic crest TADs may contribute to molar intrusion during distalization, enhancing vertical control [12].

Mandibular Molar Distalization

Three studies evaluated mandibular molar distalization [7,10,14]. The strongest evidence came from a prospective comparative study showing significantly greater distalization efficiency with microimplant anchorage compared with aligners alone [7]. However, improvements were primarily observed at the crown level, while root movement remained limited.

Comparative studies also demonstrated improved preservation of anterior anchorage and reduced molar tipping with skeletal anchorage [7,14]. Nevertheless, one retrospective study found no significant increase in distalization magnitude, suggesting that anatomical limitations may restrict treatment efficiency even when TADs are used [10].

Vertical Correction

Evidence for TAD-supported vertical correction was limited to two case-based reports [1,11]. Successful treatment of both severe anterior open bite and deep bite was achieved using miniscrew-assisted intrusion mechanics combined with CAT. Reported outcomes included substantial overbite correction, favorable vertical skeletal changes, and short-term treatment stability. However, the evidence remains limited to proof-of-concept observations.

Anterior Retraction

One case report described extraction-space closure and anterior retraction using miniscrew anchorage and clear aligners [3]. Significant incisor retraction and profile improvement were achieved with minimal posterior anchorage loss, demonstrating the potential of skeletal anchorage to support maximum-anchorage mechanics. However, the appliance design differed substantially from contemporary aligner systems.

Transverse Correction

One study reported successful correction of a severe unilateral scissor bite using CAT combined with multiple miniscrews [9]. Marked occlusal improvement and stability at two-year follow-up were observed. Although the underlying skeletal discrepancy remained unchanged, the case demonstrated the feasibility of TAD-assisted transverse correction in complex adult malocclusions.

Impacted Canine Management

Two case series investigated impacted canine traction using TADs and CAT [2,4]. In all reported cases, skeletal anchorage was used to facilitate canine eruption while aligners were employed for space creation, alignment, or finishing. Functional eruption was achieved without major periodontal complications or TAD failures. However, the evidence was limited to a small number of carefully selected cases.

Surgery-First and Complex Hybrid Treatment

One case report described a fully digital surgery-first orthognathic approach in which TADs were used adjunctively during postoperative CAT to correct residual dental discrepancies [5]. Because the primary treatment effects resulted from surgery, this study provides limited evidence regarding the independent contribution of TADs to aligner treatment outcomes.

Adverse Events

Adverse events were infrequently reported and were not systematically assessed in any study. The most commonly reported complication was miniscrew failure, which was successfully managed by repositioning the device [6,9]. No severe complications, such as nerve injury, sinus perforation, or major infection, were reported. Several studies specifically noted the absence of root resorption or periodontal deterioration [1,2,5]. However, the lack of standardized safety assessment limits conclusions regarding the true incidence of complications.

Overall Synthesis

The available evidence indicates that combining TADs with CAT is technically feasible across a wide range of clinical applications. The most consistent benefit is improved control of tooth movement, particularly reduced anchorage loss, molar tipping, and undesirable vertical effects [6-8,12,14]. In contrast, improvements in the magnitude of achieved tooth movement appear modest, with distalization efficiency remaining substantially below planned values even when skeletal anchorage is used [8,10,12]. Given the predominance of case reports and nonrandomized studies, the overall certainty of evidence remains low, and definitive clinical recommendations cannot currently be established.

Discussion

The integration of TADs with CAT represents an attempt to overcome several well-recognized biomechanical limitations of aligners. While CAT has demonstrated effectiveness for alignment, leveling, and mild-to-moderate tooth movements, the predictability of complex movements such as bodily distalization, root torque, posterior intrusion, and maximum-anchorage space closure remains lower than that observed with fixed appliances [1-8]. This discrepancy arises because aligners primarily deliver force through surface contact with the crowns of teeth, often producing tipping moments rather than true bodily translation. Consequently, planned digital movements may not be fully expressed clinically, particularly when large anteroposterior corrections are required [6-8,12].

The use of skeletal anchorage modifies this biomechanical environment by introducing a stationary anchorage unit that is independent of the dentition. In the studies included in this review, TADs were most commonly used to support distalization mechanics through elastics, power chains, or auxiliary traction systems [6-8,10,12,14]. Across these investigations, the principal advantage of TAD anchorage was not necessarily a substantial increase in the magnitude of achieved tooth movement, but rather improved control of unwanted side effects, including anchorage loss, molar tipping, and vertical extrusion [6-8,12,14]. These findings are consistent with established orthodontic principles, demonstrating that skeletal anchorage primarily improves force direction and anchorage preservation rather than eliminating biological and anatomical limitations to tooth movement.

Another important consideration is the interaction between aligner biomechanics and auxiliary force systems. Unlike conventional fixed appliances, CAT requires maintenance of the aligner fit throughout treatment. The presence of miniscrews, elastics, cantilevers, or power chains may necessitate modifications to aligner design and staging protocols [2,4,5]. In several reports, successful treatment depended on hybrid approaches that combined aligners with sectional wires, elastic traction, or staged surgical exposure procedures [2,4,13]. These observations suggest that successful integration of TADs with CAT requires careful digital treatment planning and an understanding of how auxiliary forces interact with aligner-generated forces.

The available evidence also highlights the potential versatility of TAD-assisted CAT beyond distalization mechanics. Case-based reports demonstrated successful application in anterior open-bite correction, deep-bite treatment, impacted canine traction, transverse scissor-bite correction, and extraction-space closure requiring maximum anchorage [1-5,9,11,13]. Although these reports provide only low-level evidence, they illustrate the broad range of clinical situations in which skeletal anchorage may expand the capabilities of aligner therapy. Nevertheless, clinicians should recognize that many of these applications remain supported primarily by proof-of-concept evidence rather than high-quality comparative research.

From a clinical perspective, patient compliance remains an important factor even when skeletal anchorage is employed. While TADs reduce dependence on dental anchorage, treatment success continues to rely on adequate aligner wear and adherence to prescribed elastic protocols when elastics are incorporated into treatment mechanics [6,8,10]. The limited reporting of compliance across studies represents an important evidence gap because variability in aligner wear may influence treatment efficiency and confound interpretation of treatment outcomes.

Finally, the absence of standardized outcome measures remains a major obstacle to evidence synthesis in this field. Studies varied substantially in their use of cephalometric analyses, digital model superimpositions, cone-beam computed tomography measurements, and qualitative clinical assessments [6-8,10,12,14]. Future investigations should adopt standardized three-dimensional outcome metrics and prospective study designs to facilitate comparison across studies and improve understanding of the true clinical benefits of combining TADs with CAT.

Limitations

This review has several limitations. First, the available evidence was limited by the predominance of case reports and case series, with no randomized controlled trials identified. Second, substantial heterogeneity existed across studies in patient characteristics, clinical indications, aligner systems, TAD protocols, and outcome assessment methods, precluding quantitative meta-analysis. Third, outcome measurements were not standardized, limiting direct comparison between studies. Fourth, publication bias cannot be excluded, as successful and innovative cases may be more likely to be reported than unsuccessful treatments. Fifth, patient compliance with clear aligner wear and adjunctive mechanics was rarely assessed despite its potential influence on treatment outcomes. Finally, this review was not prospectively registered, which represents a methodological limitation. Consequently, the findings should be interpreted cautiously, and higher-quality prospective studies are needed to strengthen the evidence base.

## Conclusions

The combination of TADs and CAT appears to be a feasible approach for managing selected orthodontic movements that may be challenging with aligners alone. The available evidence suggests that skeletal anchorage may enhance control of tooth movement and reduce undesirable treatment effects, particularly in cases requiring increased anchorage demands. However, current evidence remains limited in both quantity and quality, preventing definitive conclusions regarding effectiveness and optimal clinical indications. At present, TADs should be considered a potentially useful adjunct to CAT rather than a predictable solution for overcoming all biomechanical limitations of aligners. Future high-quality prospective research using standardized outcome measures and long-term follow-up is needed to better define the role of this combined treatment approach in contemporary orthodontic practice.
